# Development and Validation of an RP-HPLC-PDA Method for Determination of Paracetamol, Caffeine and Tramadol Hydrochloride in Pharmaceutical Formulations

**DOI:** 10.3390/ph14050466

**Published:** 2021-05-15

**Authors:** Fernando J. Pereira, Aida Rodríguez-Cordero, Roberto López, Luis C. Robles, A. Javier Aller

**Affiliations:** 1 Department of Applied Chemistry and Physics, Area of Analytical Chemistry, Faculty of Biological and Environmental Sciences, Campus de Vegazana, s/n, University of León, E-24071 León, Spain; arodrc03@estudiantes.unileon.es (A.R.-C.); lcrobg@unileon.es (L.C.R.); 2 Department of Applied Chemistry and Physics, Area of Physical Chemistry, Faculty of Biological and Environmental Sciences, Campus de Vegazana, s/n, University of León, E-24071 León, Spain; rlopg@unileon.es

**Keywords:** paracetamol, caffeine, tramadol, HPLC, photodiode-array

## Abstract

Paracetamol (acetaminophen) (PAR), caffeine (CAF) and tramadol hydrochloride (TRA) are important drugs widely used for many clinical purposes. Determination of their contents is of the paramount interest. In this respect, a quick, simple and sensitive isocratic RP-HPLC method with photodiode array detection was developed for the determination of paracetamol, caffeine and tramadol in pharmaceutical formulations. An improved sensitive procedure was also evolved for tramadol using a fluorescence detector system. A C_18_ column and a mobile phase constituted by methanol/phosphate were used. LODs were found to be 0.2 μg/mL, 0.1 μg/mL and 0.3 μg/mL for paracetamol, caffeine and tramadol hydrochloride, respectively, using photodiode-array detection. Alternatively, LOD for tramadol decreased to 0.1 μg/mL with the fluorescence detector. Other notable analytical figures of merit include the linear concentration ranges, 0.8–270 μg/mL, 0.4–250 μg/mL and 1.0–300 (0.2–40) μg/mL, for the same ordered analytes (including the fluorescence detector). The proposed method was successfully applied for the quantitative determination of the three drugs in tablet dosage forms.

## 1. Introduction

Currently, drug analysis is of the greatest concern in analytical chemistry, especially in the pharmaceutical industry since it can help to select the dosage form by studying the stability of the active compound and to identify the impurities in pharmaceutical formulations [1]. However, analytical determinations of these compounds not only apply to the drugs manufacturing process but also to forensic science and for quantification of prohibited substances in doping [2] or drug abuse issues [3]. In addition, the growing demand for these compounds [4], together with the misuse that the population makes of them, cause wide pollution of waters and soils [5]. Consequently, knowing the concentration levels of these compounds in the environment, the body fluids and pharmaceutical formulations, could help in optimizing their use, also avoiding harmful effects they can cause [6].

One of the most used active ingredients worldwide is paracetamol (*N*-(4-hydroxyphenyl)acetamide or acetaminophen). In commercial pharmaceutical formulations, this antipyretic drug can be found alone or mixed with other substances, either stimulants, such as caffeine (1,3,7-trimethylpurine-2,6-dione), or analgesics, such as tramadol hydrochloride ((±)-cis-2-(dimethylaminomethyl)-1-(3-methoxyphenyl)cyclohexanol hydrochloride). These combinations are widely used in clinical treatments to overcome pain, headache, fever and other ailments in humans [7,8,9]. Different analytical methods have been used for the separation/determination of paracetamol [10,11,12,13], caffeine [14,15,16], and tramadol hydrochloride [17,18], or even mixtures of them, such as paracetamol and caffeine [19,20,21,22,23,24] or paracetamol and tramadol hydrochloride [25,26,27,28,29,30,31]. In the majority of these works, a C_18_ column for high performance liquid chromatography (HPLC) separation has been the most largely used stationary phase, together with a mobile phase mainly based on acetonitrile at acidic pH and a photodiode-array (PDA) [13,21,22,25,27] or mass spectrometric detector [32,33,34]. Moreover, simultaneous determination of paracetamol, caffeine and tramadol has been possible by gas chromatography–mass spectrometry (GC–MS) using a column Macherey-Nagel Optima 5MS Accent (30 m × 0.25 mm) and injection temperature at 270 °C [34]. Similarly, the same three analytes, together with other compounds, have been determined by desorption electrospray ionization–mass spectrometry (DESI–MS) [32]. Nonetheless, additional analytical instrumentation based on cyclic voltammetry has also provided successful procedures for the determination of paracetamol [35,36], and paracetamol and caffeine [37] and paracetamol, caffeine and tramadol [38] in solid formulations.

Some of the above methodologies use sophisticated or expansive instrumentation, they are methodologically time-consuming, and/or suffer from different interfering effects. This work aimed to develop a simple facile, rapid, practical and sensitive reversed phase (RP)-HPLC-PDA method for the separation/determination of paracetamol, caffeine, and tramadol hydrochloride in bulk drug and pharmaceutical dosage forms using the most commonly employed C_18_ column and a methanol-based mobile phase. The proposed procedure includes a simple and robust methodological approach, also working under mild chemical conditions, easily accessible to any general laboratory. The main reason behind the incorporation of the methanol-based mobile phase in the developed procedure is the lack of interpretation of its behavior, also facilitating the understanding of the separation mechanism and simplifying the development of the analytical method. An analytical comparison of the determination of tramadol was also performed using a more sensitive fluorescence detection system.

## 2. Results and Discussion

### 2.1. Preliminary Studies

Before assaying the chromatographic separation of the drugs, UV–Vis absorption spectra of the three analytes were carried out individually (Figure S1) at pH 5.0. Figure S1 shows that the wavelength of maximum absorption differs slightly for each analyte. Consequently, as the PDA detector used in the HPLC system does not allow selection of the best absorption wavelength for each analyte in the same analysis whether they are simultaneously present in a sample, a compromise selection was possible at 210 nm. Nonetheless, we can make minimal variations of these analytical wavelengths (Figure S1) for individual determinations of the analytes, thus improving slightly the analytical characteristics of the developed method. The fluorescence spectrum of tramadol was also evaluated, from which the best emission wavelength was located at 296 nm, as already used in other works [39].

### 2.2. Analytical Method Optimization

For an appropriate experimental and theoretical evaluation of the chromatographic process, it is convenient to previously optimize the effect of the following experimental parameters: mobile phase composition (% MeOH), pH, phosphate concentration and flow rate. Nonetheless, for a better understanding of the possible retention mechanism, comments about the molecular structure of the three analytes are included in Supplementary Materials.

#### 2.2.1. Effect of the Mobile Phase Composition

**Figure 1:**
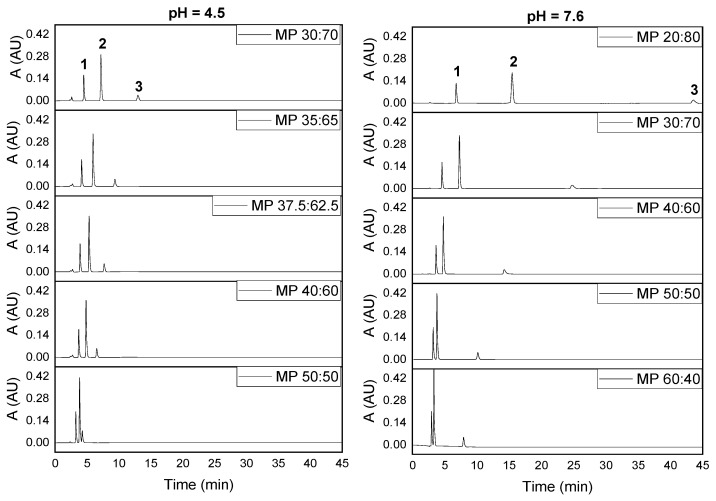
Chromatograms at basic and acidic pHs for several methanol/phosphate ratios (% MeOH/% phosphate solution, *v/v*) of the mobile phase (MP) (1: paracetamol; 2: caffeine; 3: tramadol). Paracetamol (50 μg/mL), caffeine (50 μg/mL) and tramadol (50 μg/mL). (A: absorbance; AU: arbitrary units).

Figure 1 shows the effect of the mobile phase (MP) composition (methanol/phosphate ratio or volume fraction of methanol, % MeOH) in the separation of the three analytes at two pH values. From Figure 1, we can see that the separation process was more efficient as the % MeOH of the mobile phase decreased, probably due to the concurrence of several effects. First, there is a direct relationship between the retention times and the molecule size (number of hydrophobic moieties), where paracetamol, the smallest one, eluted first. However, tramadol, containing longer bonds (single C–C bonds), eluted later than the other two drugs, which have more unsaturation centers (shorter bonds). Further, the phosphate ions, neutralizing the tramadol charge through the ion-pairing effect, made retention easier for the smaller methanol/phosphate ratios. On the other hand, methanol, a less polar solvent than water, hindered retention due to the formation of hydrogen bridges with the residual silanol groups [40]. Hence, by decreasing % MeOH under acidic pHs, the surface silanols are much more masked by protons, while at basic pHs, silanols are largely deprotonated, facilitating retention of the drugs. These effects were particularly noted for tramadol, heavily protonated at acidic pHs, but not for paracetamol and caffeine (neutral molecules). This behavior can also be viewed through the retention time and peak spacing, Δ*t*, which decreased exponentially as % MeOH increases (Figure S2), particularly under basic conditions (pH 7.6) as long as tramadol was involved. However, the peak spacing for the pair caffeine-paracetamol showed the same behavior at both acidic and basic pHs, due to the similar hydrophobic behavior of these analytes. On the other hand, the peak height grew slightly with % MeOH, while the peak area was nearly constant, as seen in Figure S3. Consequently, the peak width decreased for high contents of % MeOH (Figure S4), indicating changes in the kinetic of the retention process. A good separation of the three analytes was possible for the 40% MeOH (MP 40:60 *v/v*) and consequently, we used this mobile phase composition in the following assays.

#### 2.2.2. Effect of pH of the Mobile Phase at 40% (*v/v*) MeOH

**Figure 2:**
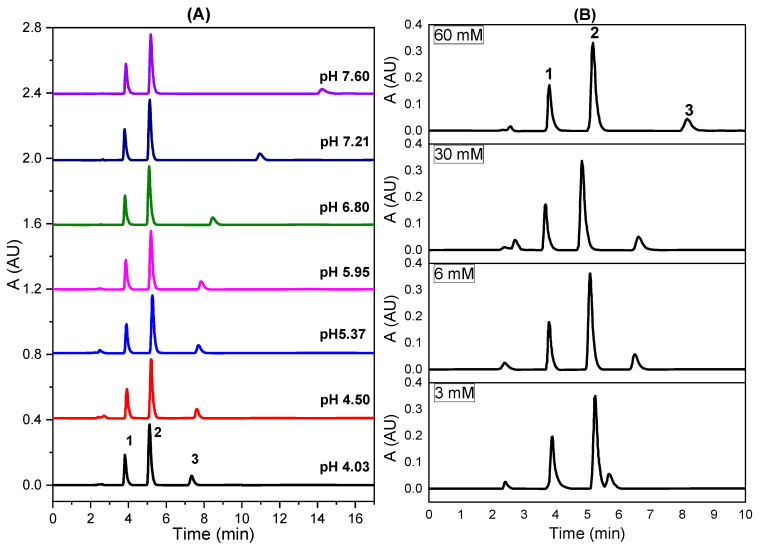
Chromatograms showing the separation of the three drugs using the selected 40% MeOH of the mobile phase at various (**A**) pH values and (**B**) phosphate concentrations (pH 4.5). Molarity values included in the picture (**B**) correspond to the phosphate concentration in the mobile phase (1: paracetamol; 2: caffeine; 3: tramadol). Other parameters as in Figure 1.

We spread the effect of pH over a wider range (Figure 2A). Figure 2A shows that the retention of tramadol increased strongly with pH, especially under basic conditions, while pH did not really affect the retention process of paracetamol and caffeine. The behavior of tramadol is explained by its basic character because at acidic pHs tramadol is mainly present as the cationic form, since the N atom is largely protonated forming a tertiary ammonium salt. However, paracetamol and caffeine show neutrality in the whole pH range studied. On the other hand, the peak width for tramadol grew with pH, but not for paracetamol and caffeine (Figure S4).

#### 2.2.3. Effect of pH of the Phosphate Concentration

The effect of the phosphate concentration on the retention of the analytes at pH 4.5 (Figure 2B) showed the same pattern than that obtained for pH (Figure 2A). Thus, the retention of tramadol increased strongly with the phosphate concentration probably involving the ion-pairing effect, because at pH 4.5, H_2_PO_4_^−^ is the predominant phosphate species (Figure S5), while at pH 7.6, two phosphate ions (H_2_PO_4_^−^ and mainly HPO_4_^2−^) coexist in solution (Figure S5). The peak spacing between tramadol and the couple paracetamol/caffeine grew with the phosphate concentration, probably a result of an increase in the ionic strength of the mobile phase. In this way, the effective concentration (activity) of methanol decreases, also suppressing the silanophilic contributions to retention [41]. On the other hand, the delay of the retention times resulting from the increase in surface tension, and consequently from the entropy of the analyte–solvent interface would be less plausible because this effect should be noted in the same way for all three analytes.

#### 2.2.4. Effect of the Flow Rate of the Mobile Phase

Figure S6 shows that a more efficient interaction between the analytes and the stationary phase made the retention time longer for lower flow rates, while the peak width and peak spacing decreased for higher flow rates (shorter retention times). The flow rate changed the retention kinetics because the peak area grew faster than the peak height (Figure S7). Thus, the peak area increased linearly with the inverse of the flow rate (Figure S8), whilst the peak height stabilized below 0.8 mL/min.

**Figure 3:**
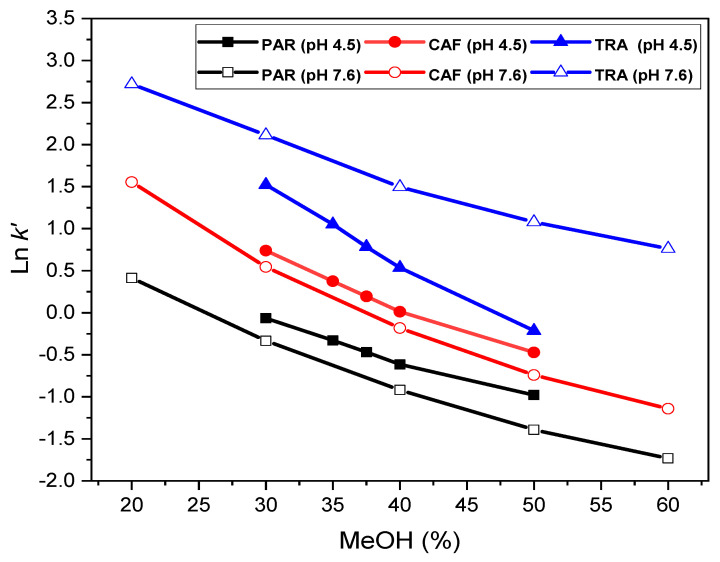
A plot of *Ln k’* vs. *% MeOH* for paracetamol, caffeine and tramadol (peaks 1, 2 and 3, respectively as Figure 1).

The retention (capacity) factor, *k’*-value, decreased as the analyte concentration increased (and consequently the peak area) (Figure S9). This behavior of the retention process results from overloading of the silanol groups [42] and shows a typically Langmuirian model. Similarly, the *k’*-value for tramadol grew with pH (Figure S10A), as befits a basic (cationic) character. Contrarily, *k’* for paracetamol and caffeine showed no variation with pH, representing a more typical neutral character in the pH range covered [41,43]. The same behavior was noted with the phosphate concentration, probably due to neutralization of the highly compensated positive charge supported by the tertiary amine of the tramadol molecule [44]. This behavior would be the result of the hydrophobic character involved in the retention process, largely modulated by the nature of the organic modifier (MeOH). Hence, the plot of *Ln k’* vs. *% MeOH* (Figure 3) showed slightly curved lines for paracetamol and caffeine, owing to their very weak basic character [45]. Therefore, a quadratic model fit better the experimental data, mainly at basic pHs (Table 1), although the quadratic terms took values very close to zero. Alternatively, at acidic pHs, the plots showed better linearity, but covering a narrower % MeOH range and with lines relatively close to each other, accounting for most similar retention properties of the analytes under acidic conditions. Linearity relates to hydrophobic compounds, according to the solvophobic theory, but usually with slight disturbances due to overlapping π–π interactions. The plot of *Ln k’* vs. *% MeOH* for tramadol showed a more negative slope at pH 4.5 than at basic pH (Figure 3), indicating greater interaction with the silanol groups at basic pHs. On the other hand, the slopes for paracetamol and caffeine are very similar at acidic and slightly basic, confirming again the no influence of pH on their retention.

The chromatograms obtained in this work show asymmetric peaks, with *b* > *f* (Table S1). The asymmetry factor, *AF* = *b/f*, and the tail factor, *TF* = (*b* + *f*)/2*f*, showed values above unity, which suggests strong retention. Both *b* and *f* grew with the retention time (*w* grew), but *f* grew faster than *b*, which means that the *AF* parameter decreases similarly for longer retention times. In the same way, as a general behavior, *AF* decreased for increased phosphate concentration, pH and % MeOH because the three experimental variables delay in some way the retention times. Nonetheless, slight alterations were noted with % MeOH at basic pHs (Figure S10B), probably because the acidity pK_a_ changed with the methanol/phosphate ratio [46]. However, for low values of % MeOH and at pH 7.6, the parameter *b* grew faster than *f* (and consequently *AF* increased), which means that the three analytes were released slowly but in different extension by the stationary phase. On the other hand, *AF* also grew with the analyte concentration, probing again a Langmuir-type behavior of the retention process. Worth noting that tailing and fronting peaks result from Langmuir and anti-Langmuir behaviors, respectively. Assuming asymmetric peaks (*b* ≠ *f*), the effective number of plates, N*, showed a direct logical relationship with the retention times (Table S1). On the other hand, the most coherent values of the column resolution *Ra’* resulted from averaging the efficiency ($\bar{N}$) and the averaged capacity factor ($\bar{k^{\prime }}$) (Table S1), which represents a more reasoning situation, since characteristics from analytes were combined.

### 2.3. Validation of the Analytical Method

After optimization of the most important experimental parameters, we proposed an analytical procedure whose optimal characteristics were included in Section 3.3 and briefly described here. Mobile phase containing a methanol/phosphate ratio of 40/60 *v/v*, pH 4.5, phosphate concentration of 6 mM in the finally analyzed sample, flow rate pf 1 mL/min, working at room temperature, detection wavelength at 210 nm for the three analytes and injection volume of 20 µL. For better reliability of the best analytical method, it is recommended to carry out validation assays, checking the following analytical characteristics: linearity range, accuracy, precision, the limit of detection (LOD), the limit of quantification (LOQ), robustness/ruggedness and specificity/stability [47]. Furthermore, to improve the analytical capabilities, the same evaluation of the above analytical characteristics was studied replacing the PDA detector with a fluorescence detection system.

#### 2.3.1. Linearity Range

**Figure 4:**
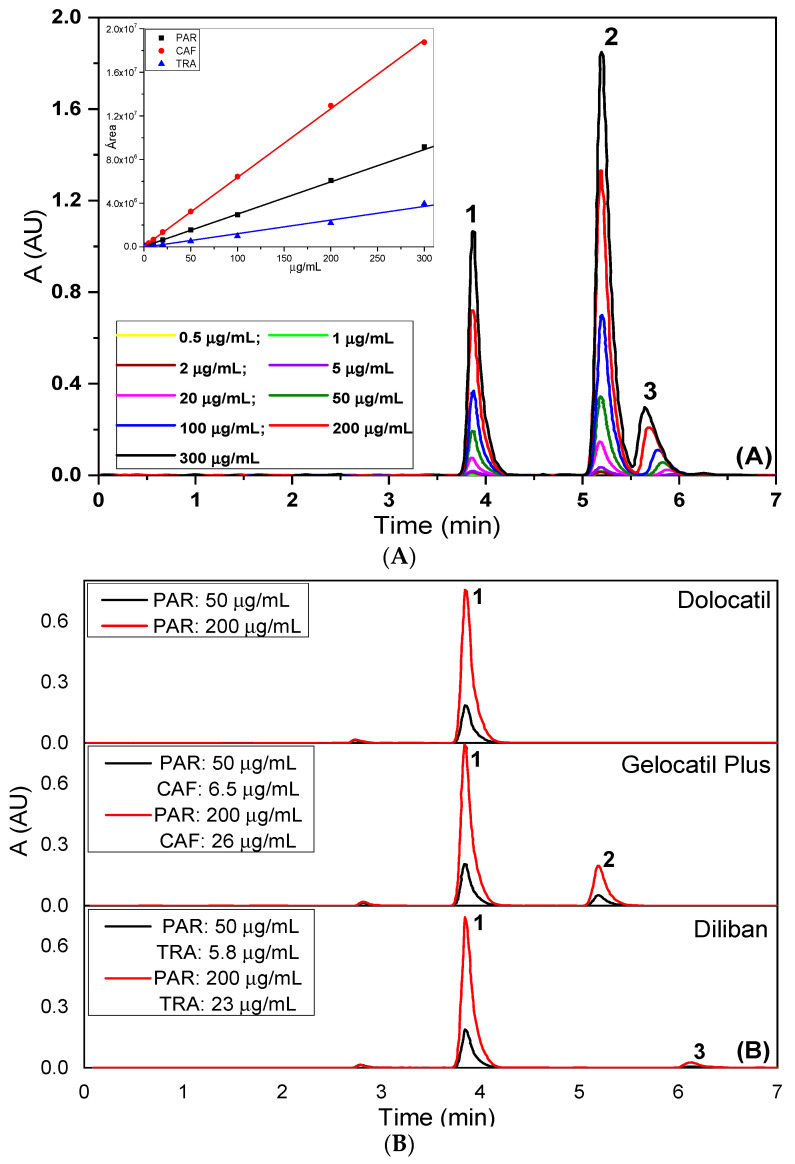
Chromatograms obtained for various concentrations of paracetamol, caffeine and tramadol (peaks 1, 2 and 3, respectively) from (**A**) standard solution and (**B**) real samples. The inset in (**A**) represents the corresponding straight lines.

The linearity range of the proposed method was established using nine aliquots of the standard stock solutions, transferred to a series of 10 mL volumetric flasks, and adjusting the volume to the mark with the mobile phase. Final concentrations between 0.5 and 550 μg/mL for each analyte were covered. Three replicates per each concentration of the above solutions were injected and peak areas and heights were reported. A calibration line of peak area vs. concentration was established and regression equations were performed with high coefficients of determination (Figure 4A, Table 2). Figure 4B shows the corresponding chromatograms related to the three real samples.

#### 2.3.2. Accuracy

The accuracy of the developed method was evaluated using a minimum of nine determinations by performing recovery studies by the standard addition method, at three concentration levels covering the specified range. Furthermore, three pharmaceutical commercial preparations, Dolocatil^®^ (88.7% *w/w* paracetamol), Diliban^®^ (81.4% *w/w* paracetamol, 9.4% *w/w* tramadol hydrochloride) and Gelocatil-Plus^®^ (74.7% *w/w* paracetamol, 9.6% caffeine), were checked as real samples. The mean recoveries were found in the range of 98.47–101.10% (Table 2).

#### 2.3.3. Precision

The precision of an analytical procedure expresses the closeness of agreement between a series of measurements obtained from multiple sampling of the same homogenous sample under the recommended conditions. The values were reported as relative standard deviation (RSD, %), being all of them lower than 4% (Table 2).

#### 2.3.4. Limit of Detection (LOD)

LOD is the lowest concentration of analyte that can be qualitatively detected, but not quantified, by the analytical method. In chromatography, it is common to calculate the limit of detection as the injected amount that results in a peak height at least twice or three times the baseline noise level (usually S/N ratio 3). However, LODs in this work (Table 2) were peak area-based calculated following the IUPAC recommendations, according to the following equation,

(1) $$LOD=\frac{3.29\;S_{y/x}}{m}\;\sqrt{\frac{1}{3}+\frac{1}{n}+\frac{{\bar{x}}^{2}}{S_{x/x}}}$$

where *m* is the slope of the calibration straight line, *n* represents the number of calibration points involved in the regression procedure, $\bar{x}$ is the mean concentration of all points used in calibration, S_x/x_ is the residual standard deviation being equal to: $S_{x/x}=\sum_{i=1}^{n}{\left(x_{i}-\bar{x}\right)}^{2}$, *x_i_* is the *i*-th point of the concentration coordinate and *S_y/x_* represents the error in regression, given by the squared deviation of ${\hat{y}}_{i}$, calculated in regression, from *y_i_*, measured in calibration experiment:

(2) $$S_{y/x}=\sqrt{\frac{1}{n-2}\;\overset{n}{\underset{i=1}{\sum }}{\left(y_{i}-{\hat{y}}_{i}\right)}^{2}}=\sqrt{\frac{1}{n-2}\;\overset{n}{\underset{i=1}{\sum }}{\left(y_{i}-a-m\;x_{i}\right)}^{2}}\;$$

where *a* and *m* relate to the general straight-line calibration model, *y_i_* = *a* + *m x_i_*. Good LODs were achieved in this work, compared with those found by other authors using similar techniques (Table S2).

#### 2.3.5. Limit of Quantitation (LOQ)

LOQ is the lowest concentration of analyte that can be quantitatively determined with acceptable accuracy and precision by the analytical method. In chromatography, LOQ represents the analyte concentration generating an instrument response equivalent to ten times the noise (S/N ~ 10). However, the LOQs shown in Table 2 were peak area-based calculated, according to the IUPAC recommendations, using the following equation,

(3) $$LOQ=\frac{10\;S_{y/x}}{m}\;\sqrt{\frac{1}{3}+\frac{1}{n}+\frac{{\bar{x}}^{2}}{S_{x/x}}}$$

with the same meaning as above for the parameters involved. A comparison with the LOQs found by other authors is included in Table S2.

#### 2.3.6. Robustness/Ruggedness

The robustness of a method is the ability to remain unaffected by the deliberate variations in the method parameters, such as column temperature, analytical wavelength, flow rate and mobile phase composition (% MeOH, pH, phosphate concentration). Changes of these parameters around 10% of their optimum value do not affect (<5%) the analytical signal (peak area) provided by the detector. Thus, detection wavelength was initially fixed to 210 nm and then changed to 220 nm and a series of three injections of 100% concentration were given at each detection wavelength and the % RSD was reported separately for each variable. On the other hand, the flow rate was varied between 0.8 and 1.2 mL/min and a series of three injections of 100% concentration were given at each detection wavelength and deviations (% RSD) were reported (Table 2). The peak area decreased exponentially with the flow rate (grows linearly with the inverse of the flow rate) (Figure S4), but not very much for the high values of the range covered. Ruggedness is a measure of reproducibility of the results under instrument-to-instrument and analyst-to-analyst variations. The coanalyst gave six injections of 100% concentration and deviations (% RSD) in the peak areas were recorded. The global results are reported in Table 2.

#### 2.3.7. Specificity/Stability

Specificity was initially checked by comparing calibration lines using analytes standards and drugs from pharmaceutical dosage formulations. For both situations, straight lines with similar slopes (slopes ratios close to unity) were obtained for each analyte. For a better understanding of the specificity and stability of the developed method, we carried out a tentative evaluation of the potential degradation of these molecules submitting their solutions to the effect of natural light for six months at room temperature. Then, the solutions with the degradation products were analyzed under the optimized chromatographic conditions. The three drugs were determined with minor variations (<5%), which suggests a high specificity of the developed procedure since even potential degradation products do not interfere in the separation/determination of the three analytes. According to the findings from the scientific bibliography, paracetamol and tramadol show a degradation process smaller than caffeine. Caffeine is not efficiently degraded by near-UV–Vis irradiation, requiring a photocatalytic approach for a more efficient process [48]. Notwithstanding, under stronger oxidation conditions, several degradation compounds are possible to find [49,50,51,52].

Other chromatographic parameters are included in Table S1.

### 2.4. Structural Characteristics of the Analytes

Paracetamol is made up of a benzene ring core, joined in the *para*-(1,4) pattern to two activating groups, one hydroxyl group and the nitrogen atom of an amide group (acetamide or ethanamide). Consequently, the paracetamol molecule constitutes an extensively conjugated system, including the lone pairs on the hydroxyl oxygen, nitrogen atom and carbonyl oxygen, together with the benzene π cloud and the *p* orbital on the carbonyl carbon (Figure S11). As a result of the presence of two electron-donating groups, the benzene aromatic ring of this system shows more reactivity than usual, thus facilitating π–π interactions. All positions on the benzene ring are equally activated because the substituents are *ortho-para*-directing. The conjugation also greatly reduces the basicity of the oxygen (O11) and nitrogen (N13) atoms (Figure S12), thus increasing the hydroxyl acidity (pK_a_ = 9.38), although altered with the mobile phase composition [53] through delocalization of charge developed on the phenoxide anion. This means that paracetamol is in its neutral form below the maximum pH value covered (pH 7.6). Only above pH 9.38, the ionic (deprotonated) form starts to take relevance (Figure S5) [54,55,56]. The acetamide group can undergo hydrolysis and the phenol group acid/base reactions. In general, the presence of amide and hydroxyl groups acts as hydrogen bond donors whereas the carbonyl and hydroxyl groups act as hydrogen bond acceptors within the molecule. As a result, the paracetamol molecule can form hydrogen bridges, intramolecularly through the OH and NH groups, particularly >C=O(16)…..H-O(11) and (13)N-H…..N(13), but also intermolecularly, more plausibly with methanol, because the solubility of paracetamol in methanol is higher than in water [57]. At very acidic pHs (pH < 0.14), protonated paracetamol in the ${\mathrm{NH}}_{2}^{+}$ group could theoretically coexist with the neutral form. However, possible potential protonation in the C=O group is less plausible to occur because the O atom is part of a conjugated system. On the other hand, at very low pHs, the formation of dimers (poly-condensation process) is facilitated.

The geometry of the caffeine molecule represents a large planar conjugated system incorporating as the most important functional groups: one tertiary amine and two amide groups (Figure S11). Other characteristic groups include methyl (-CH_3_), carbonyl (>C=O), alkene (-C=C-) and imine (>C=N-). The six-membered pyrimidinedione can exist in an aromatic form where both amide nitrogens have formed double bonds to respective adjacent carbonyls. Therefore, caffeine does indeed exist primarily as an ionized resonant form, with two partial positive charges on the two nitrogen atoms and two partial negative charges on the two exocyclic carbonyl oxygens, but for an overall neutral molecule. The basic imidazole nitrogen likely has the largest effect on the pH. It is weakly basic (pK_a_ of conjugate acid = 0.6) requiring strong acid to protonate it. Thus, at very acidic pHs (pH < 0.6), it would be protonated on the N(9) atom (Figure S12), supporting a positive charge on N(9) very stabilized by the conjugated system of the molecule (Figure S11). Under this very strong pHs, the caffeine molecule can interact with negatively charged species or lone pair electrons. However, caffeine is not protonated in the pH range covered in this work. Caffeine is a polar molecule due to the electronegativity difference between the carbon–oxygen and carbon–nitrogen single polar covalent bonds, undergoing London dispersion and dipole–dipole intermolecular interactions.

Tramadol contains a planar benzene ring with delocalized 6π electrons (aromaticity), which is largely activated by the strong +*M* effect of the methoxy group (–OCH_3_) (Figure S11). Furthermore, it is joined to a cyclohexanol ring, which also contains in *ortho*-position a dimethylamino methyl group {–CH_2_N(CH_3_)_2_} with an electron-rich nitrogen atom. The molecule is relatively large and sufficiently planar to block a large surface area on the stationary phase, thus favoring its retention. Nonetheless, it also facilitates interaction with other molecules, such as methanol. The tramadol hydrochloride molecule used in this work is protonated on the N atom (pK_a_ = 9.23), which supports a very stabilized positive charge on a tertiary ammonium cation (Figure S12). Hence, at pHs higher than 9.23, the tramadol molecule predominates in neutral form, while at pHs below 9.23 coexists with the protonated form.

## 3. Materials and Methods

### 3.1. Chemicals and Reagents

Methanol (MeOH) (Purity > 99%, Labscan Limited, Dublin, Ireland) was used as an organic modifier of the mobile phases. Phosphate buffers were prepared in Milli-Q water (18 MΩ cm) by taking the appropriate amount of orthophosphoric acid (H_3_PO_4_) (1.71 kg/L, 85%), dipotassium hydrogen phosphate (K_2_HPO_4_) and/or potassium dihydrogen phosphate (KH_2_PO_4_) (Panreac, Barcelona, Spain). The buffer solutions were degassed by employing an ultra sonicator and, then, the pH was adjusted with hydrochloric acid or potassium hydroxide solutions (Merck, Darmstadt, Germany), as required. The pH values stated throughout the work correspond to the final mixture prepared.

Paracetamol, caffeine and tramadol hydrochloride (purity > 99%) were purchased from Sigma (Sigma-Aldrich, St. Louis, MO, USA). Standard stock solutions were prepared by accurately weighing 25 mg of the pure drugs, dissolved in 10 mL of the mobile phase, transferred into a 25 mL volumetric flask and made up to the mark. Commercial pharmaceutical products whose active principle is paracetamol (Dolocatil^®®^), paracetamol and caffeine (Diliban^®®^) and paracetamol and tramadol (Gelocatil-Plus^®®^) were also acquired in tablet form from a local pharmacy. These tablets were crushed to obtain a random mixture and 10 mg of this product was accurately weighed, dissolved in 50 mL of the mobile phase and transferred into a 100 mL volumetric flask. The solution was made up to the mark with the mobile phase, filtering through a 0.45 μm membrane syringe filter to prevent possible contamination from entering the column. All solutions were prepared using the Milli-Q water (18 MΩ cm) and sonicated for 15 min.

### 3.2. Instrumentation

An HPLC Alliance, Waters 2690 Separations Module (Waters Corporation, Milford, MA, USA) including controller, autosampler and thermostatic column oven was used. Chromatographic separation was carried out on a SunFire^TM^ C_18_ column, 4.6 mm × 250 mm, 5 μm from Waters, including a Rheodyne injector with 20 μL fixed loop. A Waters 996 photodiode array detector (PDA) with variable wavelength (210–400 nm) programmable UV detector was used in combination with a JASCO FP-2020 Plus fluorescence detector (Pfungstadt, Germany) for a comparative determination of tramadol (λ_exc_ = 200 nm, λ_em_ = 296 nm). Empire software (Scarborough, Western Australia) was used for data analysis. Absorbance measurements were recorded on a Thermo Spectronic Helios Alpha 9423 UVA 1002E UV–Vis double beam spectrophotometer (Thermo Fisher Scientific Inc., Waltham, MA, USA), combining a deuterium-discharge lamp for the ultraviolet (UV) wavelength range and a tungsten lamp for the visible and short wave near-infrared (SWNIR) wavelength range.

When necessary, the chromatographic peaks were digitized using GetData Graph Digitizer 2.26. The digitized chromatograms and UV–Vis spectra were processed using the Microcall Origin Software 9.0. A Bransonic sonicator, model B-5 (Soest, The Netherlands) was used for degasification of the sample solutions. A pH meter (Crison model Digit 505) was used to measure the acidity of the aqueous phase. A Mettler AE 240 semi microanalytical balance (sensitivity = 0.01 mg) was used for weighing the chemicals.

### 3.3. Chromatographic Conditions

The methodology used in this work to select the best optimization conditions followed the strategy called one-variable-at-a-time. The experimental chromatographic variables were optimized in the ranges: mobile phase composition (%MeOH/%phosphate ratio 20:80 *v/v*–60:40 *v/v*), pH (4.0–8.0), phosphate concentration (3–60 mM) and flow rate (MP) (0.4–1.2 mL/min). After optimization, the best chromatographic conditions were: mobile phase with a methanol/phosphate ratio of 40:60 *v/v*, pH 4.5, final phosphate concentration of 6 mM and flow rate of 1 mL/min (isocratic elution). Other fixed chromatographic parameters were room temperature, detection wavelength at 210 nm and injection volume of 20 μL. The addition of phosphate allows us to control the potential effect of the ionic strength of the mobile phase. In this work, we used, as analytical parameters the adjusted retention times.

When developing an analysis method for pharmaceuticals, it is necessary to assume the quality-by-design (QbD) guidelines from the International Conference on Harmonization (ICH) [58,59]. To validate the chromatographic procedure described here, suitability tests were carried out using freshly prepared stock standard solutions. The empirical data collected were calculated as the mean of at least three measurements.

## 4. Conclusions

A simple RP-HPLC-PDA separation method was developed and validated for the determination of paracetamol, caffeine and tramadol in bulk and pharmaceutical dosage forms. The proposed method is rapid, accurate and precise. The chromatographic retention times of the three analytes allow the analysis of a large number of samples in a short time. The use of the fluorescence detection system improved the sensitivity (LOD) for tramadol by a factor of four. The experimental and theoretical chromatographic parameters were critically evaluated in depth. From a practical point of view, the developed method is suitable for the routine analysis and stability studies of the three drugs. Applicability of this procedure would be very useful in the pharmacological and medicinal fields.

## Figures and Tables

**Table 1:** Parameter values and standard deviation (±SD) obtained for linear and quadratic fits of *Ln k’* vs. *% MeOH* at two pH situations. (*R*^2^ is the coefficient of determination).

pH	Coefficient	Paracetamol	*R* ^2^	Caffeine	*R* ^2^	Tramadol	*R* ^2^
Linear fit: *Ln k’* = *a* + *m B*							
7.6	*a*	1.350 ± 0.190	0.9721	2.700 ± 0.300	0.9597	3.620 ± 0.170	0.9755
	*m*	−0.054 ± 0.005		−0.067 ± 0.007		−0.050 ± 0.004	
4.5	*a*	1.260 ± 0.120	0.9818	2.478 ± 0.165	0.9805	4.070 ± 0.162	0.9909
	*m*	−0.046 ± 0.003		−0.060 ± 0.004		−0.087 ± 0.004	
Quadratic fit: *Ln k’* = *a* + *m B* + *c B*^2^							
7.6	*a*	2.272 ± 0.040	0.9999	4.070 ± 0.110	0.9993	4.390 ± 0.140	0.9979
	*m*	−0.106 ± 0.002		−0.146 ± 0.006		−0.094 ± 0.008	
	*c*	0.001 ± 0.000		0.001 ± 0.000		0.001 ± 0.000	
4.5	*a*	2.400 ± 0.400	0.9967	4.200 ± 0.300	0.9986	5.600 ± 0.500	0.9980
	*m*	−0.106 ± 0.016		−0.146 ± 0.014		−0.167 ± 0.024	
	*c*	0.001 ± 0.000		0.001 ± 0.000		0.001 ± 0.000	

**Table 2:** Analytical characteristics of the developed method (*n* = 9).

Parameters	Paracetamol (40:60) (PDA Detection)	Caffeine (40:60) (PDA Detection)	Tramadol (40:60)	
			(PDA Detection)	(Fl Detection)
Linear Range (μg/mL)	0.8–270	0.4–250	1.0–300	0.2–40
*R* ^2^	0.9987	0.9998	0.9999	0.9999
Accuracy (%)	98.47–99.85	99.97–100.08	101.10–101.10	99.96–100.91
RSD (%) (overall)	3.45	3.92	3.16	2.94
LOD (μg/mL)	0.2	0.1	0.3	0.1
LOQ (μg/mL)	0.8	0.4	1.0	0.2
Robustness/Ruggedness (%)	3.9	4.1	3.6	3.2
Specificity (%)	≥95	≥95	≥95	≥95
Stability (%)	≤5	≤5	≤5	≤5

## Data Availability

Data will be provided by request.

## Supplementary Materials

The following are available online at https://www.mdpi.com/article/10.3390/ph14050466/s1, Figure S1: UV–Vis spectra of the three analytes at the 10 μg/mL concentration in aqueous solution, Figure S2: (A) Adjusted retention time and (B) peak spacing against the composition of the mobile phase at two pH values, Figure S3: (A) peak area and (B) peak height against the composition of the mobile phase at two pH values, Figure S4: Peak base-width against the composition of the mobile phase at two pH values, Figure S5: Distribution diagram of phosphoric acid, paracetamol, caffeine and tramadol species as a function of pH, Figure S6: Chromatograms obtained for several flow rates (FRs) of the mobile phase using the selected 40% MeOH and pH 4.5 (1: paracetamol; 2: caffeine; 3: tramadol). Other parameters as in Figure 1, Figure S7: Peak area against peak height for the three analytes and five flow rate values, Figure S8: (A) Peak area and (B) peak height against the inverse of the flow rate for the three drugs, Figure S9: Capacity factor against the analyte concentration for the three drugs, Figure S10: Plots of (A) the capacity factor vs. pH and (B) the asymmetry factor vs. composition of the mobile phase at two pH values, Figure S11: Resonant structures for paracetamol (for two tautomers), caffeine (neutral and protonated forms) and tramadol (protonated form), Figure S12: Numeration of the carbon atoms, Table S1: Chromatographic parameters for paracetamol (50 μg/L), caffeine (50 μg/L) and tramadol (50 μg/L) based on the proposed method (mobile phase composition: 40% MeOH; pH = 4.5; phosphate concentration = 6 mM; flow rate = 1.0 mL/min; dead time, *t_o_* = 2.431 min). (*) P: paracetamol; C: caffeine; T: tramadol, Table S2: A comparison of the limits of detection (LOD) and quantification (LOQ) achieved by the developed method against other publications.
