# Supplementary material for: Development and Validation of an RP-HPLC-PDA Method for Determination of Paracetamol, Caffeine and Tramadol Hydrochloride in Pharmaceutical Formulations
Source: Pharmaceuticals (Basel). 2021 May 15;14(5):466. doi: 10.3390/ph14050466 (PMC8156741; doi:10.3390/ph14050466)
Supplement: Supplementary file 1 [file pharmaceuticals-14-00466-s001.zip › pharmaceuticals-1203113-supplementary.pdf]

pharmaceuticals

MDPI

# SUPPLEMENTARY MATERIALS

**Table S1.** Chromatographic parameters for paracetamol (50 µg/L), caffeine (50 µg/L) and tramadol (50 µg/L) based on the proposed method (mobile phase composition: 40% MeOH; pH = 4.5; Phosphate concentration = 6 mM; Flow rate = 1.0 mL/min; dead time, $t_o$ = 2.431 min). (*) P: paracetamol; C: caffeine; T: tramadol.

| Parameter | Equation | Analyte | | | |
|---|---|---|---|---|---|
| | 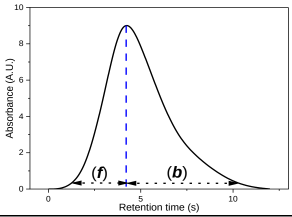 | Paracetamol (40:60) | Caffeine (40:60) | Tramadol (40:60) | Reference value |
| $b$ | | 0.179 | 0.199 | 0.256 | |
| $f$ | | 0.080 | 0.100 | 0.133 | |
| $TF$ | $= (b+f)/2f$ | 1.619 | 1.495 | 1.462 | |
| $AF$ | $= (b/f)$ | 2.24 | 1.99 | 1.92 | $T \leq 2$ |
| $t_R$ | | 3.873 | 5.228 | 6.685 | |
| $t'_R$ | $= t_R - t_o$ | 1.442 | 2.797 | 4.254 | |
| $k'$ | $= t'_R/t_o$ | 0.593 | 1.151 | 1.750 | $1 < k' < 10$ |
| $\alpha_{2/1}$ | $= \frac{(t'_R)_2}{(t'_R)_1} = \frac{k'_2}{k'_1}$ | | 1.940 (P-C)* | 1.521 (C-T)*<br>2.950 (P-T)* | $\alpha > 1$ |
| $N_a$* (asim) | $= 41{,}7 \frac{\left(\frac{t'_R}{W_{0.1}}\right)^2}{\left(\frac{f_{0.1}}{b_{0.1}} + 1.25\right)}$ | 762 | 2082 | 2818 | $N > 2000$ |
| $H$* (asim) | $= L/N_a^*$ | 0.03282 | 0.01201 | 0.00887 | |
| $R_a'$ (asim) | $= \sqrt{\frac{\bar{N}}{16}} \left[\frac{\alpha - 1}{\alpha}\right] \left[\frac{\bar{k}}{1 + \bar{k}}\right]$ | | 2.002 (P-C)* | 2.494 (C-T)*<br>3.413 (P-T)* | $R > 2$ |

pharmaceuticals

MDPI

**Table S2.** A comparison of the limits of detection (LOD) and quantification (LOQ) achieved by the developed method against other publications.

| **Paracetamol** 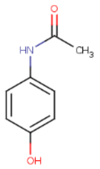 | | **Caffeine** 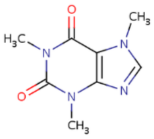 | | 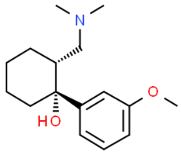 **Tramadol** | | **Detector (λ, nm)** | **Reference** |
|---|---|---|---|---|---|---|---|
| **LOD (µg/mL)** | **LOQ (µg/mL)** | **LOD (µg/mL)** | **LOQ (µg/mL)** | **LOD (µg/mL)** | **LOQ (µg/mL)** | | |
| 0.2 | 0.8 | 0.1 | 0.4 | 0.3 (0.1)* | 1.0 (0.2)* | UV-Vis (210) | This work |
| 0.03 | 0.1 | - | - | - | - | Fl* (355) | [11] |
| 0.004 | - | - | - | - | - | SWV | [12] |
| 1.0 | 10.0 | - | - | - | - | UV-Vis (210) | [13] |
| - | - | 0.100 | 0.250 | - | - | UV-Vis (215) | [14] |
| - | - | 0.152 | 0.461 | - | - | UV-Vis (272) | [16] |
| - | - | - | - | 0.7 | 0.9 | UV-Vis (218) | [17] |
| 0.467 | 1.415 | 0.752 | 2.277 | - | - | UV-Vis (λ ?) | [19] |
| 0.409 | 1.226 | 0.151 | 0.502 | - | - | UV-Vis (215) | [20] |
| 0.09 | 0.29 | 0.17 | 0.56 | - | - | UV-Vis (207) | [21] |
| 0.67 | - | 0.13 | - | - | - | UV-Vis (220) | [22] |
| 0.18 | 0.53 | 0.220 | 0.601 | - | - | UV-Vis (210) | [24] |
| 0.64 | 2.13 | - | - | 1.36 | 4.53 | UV-Vis (220) | [25] |
| 20.00 | 66.70 | - | - | 6.00 | 20.00 | GC-MS | [25] |
| 0.008 | 0.016 | | - | 1.317 | 2.634 | DPV | [26] |
| 2.589 | 7.847 | - | - | 0.479 | 1.453 | UV-Vis (281) | [28] |
| 0.035 | 0.116 | 0.019 | 0.062 | - | - | SWV | [29] |
| 35.8 | 108.5 | - | - | 4.14 | 12.54 | UV-Vis (271) | [30] |
| 0.57 | 1.74 | - | - | 0.31 | 0.95 | UV-Vis (270) | [31] |
| 0.012 | - | 0.019 | - | 0.132 | - | DPV | [38] |

*Using fluorescence detection system; SWV: Square Wave Voltammetry; DPV: Differential Pulse Voltammetry

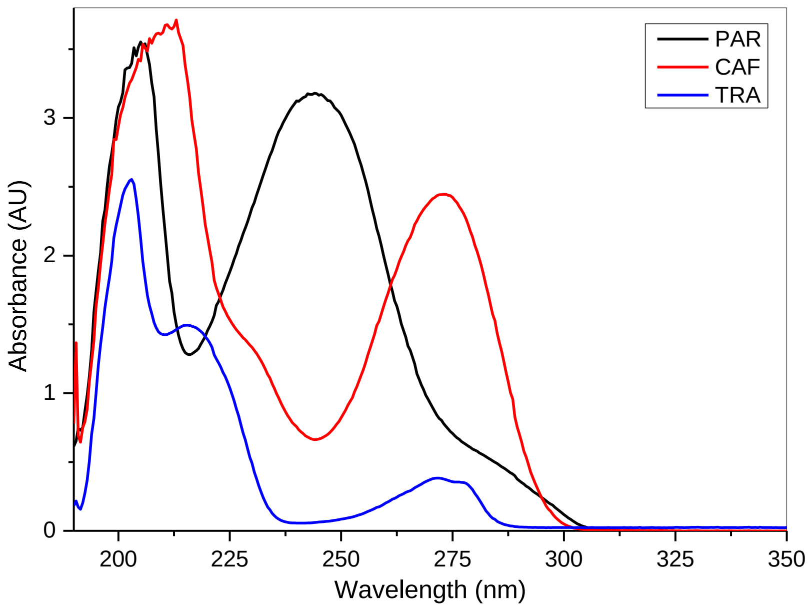


**Fig. S1.** UV-Vis spectra of the three analytes at the 10 µg/mL concentration in aqueous solution (AU: arbitrary units).

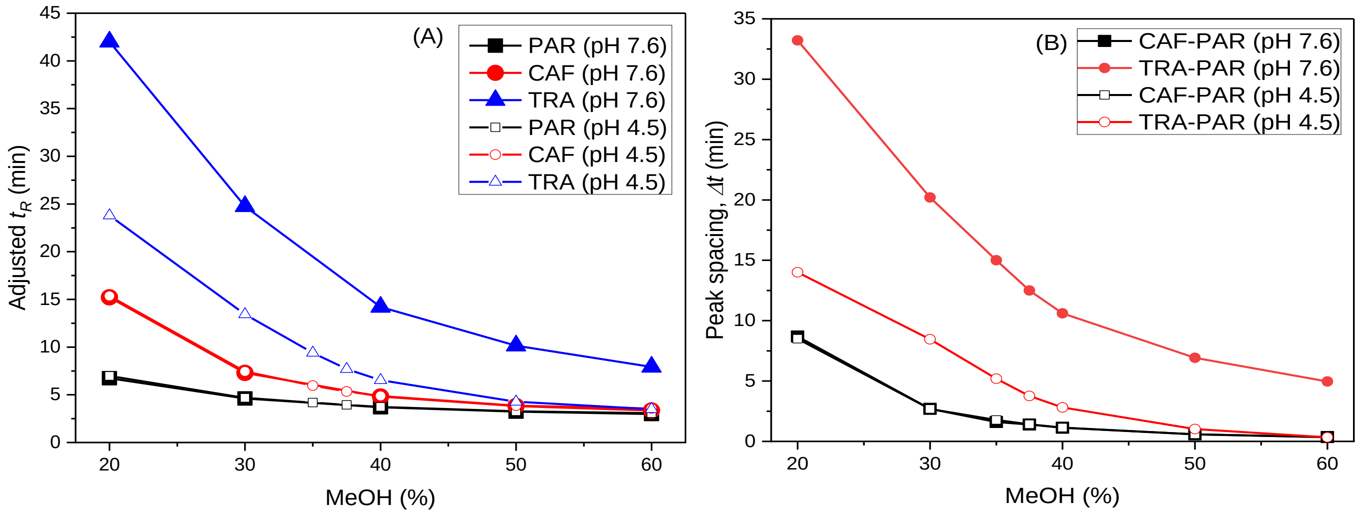


**Fig. S2.** (A) Adjusted retention time and (B) Peak spacing against the composition of the mobile phase at two pH values.

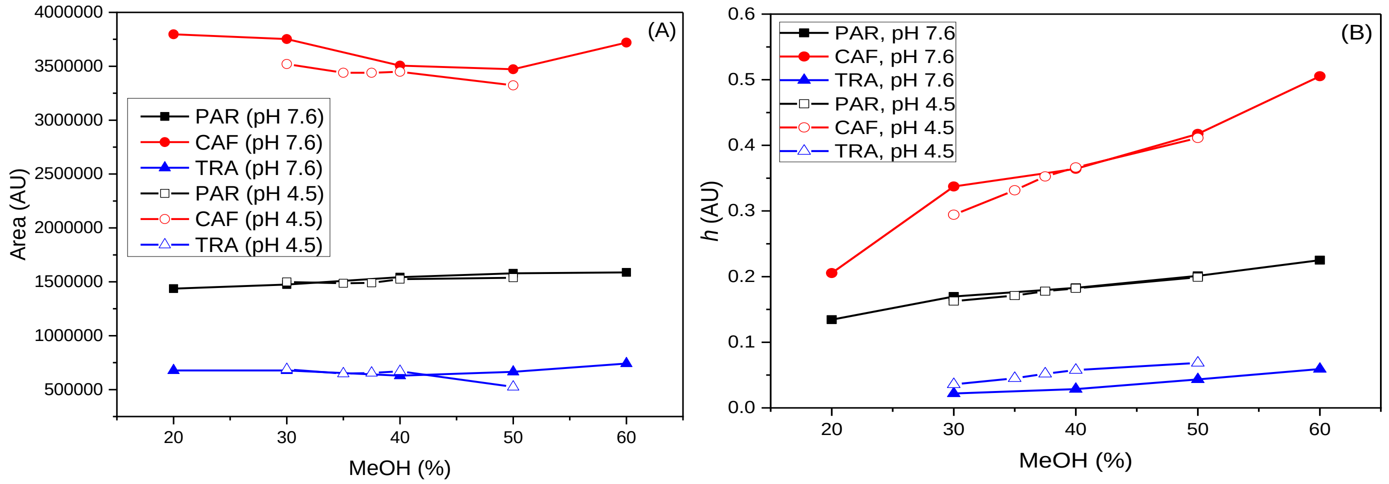


**Fig. S3.** (A) Peak area and (B) peak height against the composition of the mobile phase at two pH values (AU: arbitrary units).

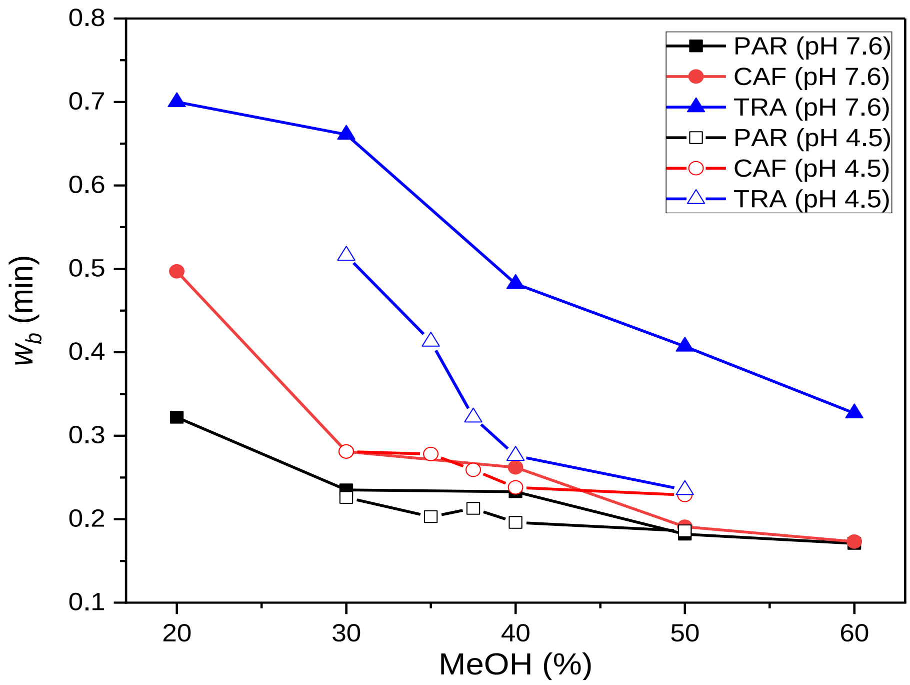


**Fig. S4.** Peak base-width against the composition of the mobile phase at two pH values.

**Phosphoric acid**

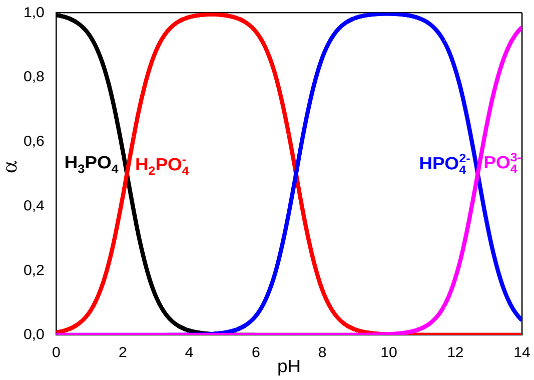


**Paracetamol**

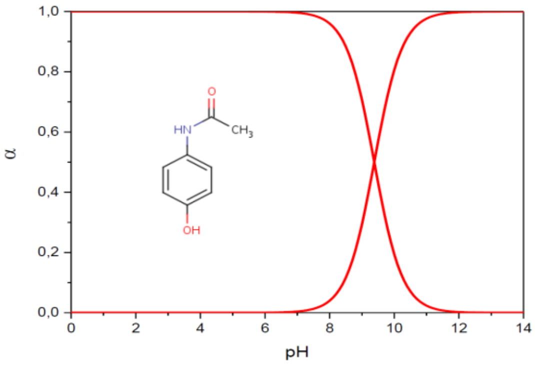


**Caffeine**

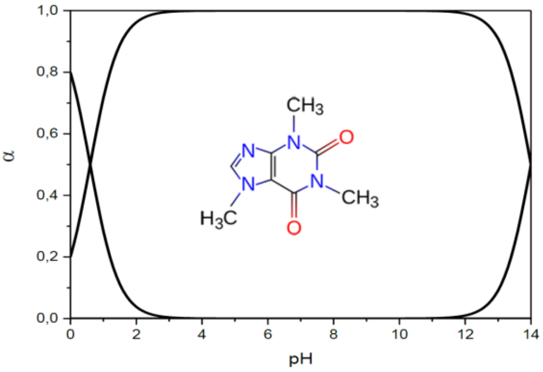


**Tramadol**

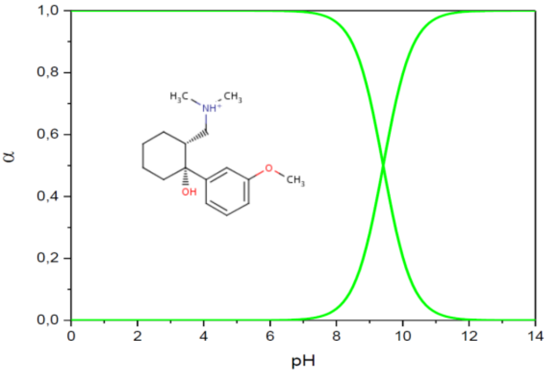


**Fig. S5.** Distribution diagram of phosphoric acid, paracetamol, caffeine and tramadol species as a function of pH.

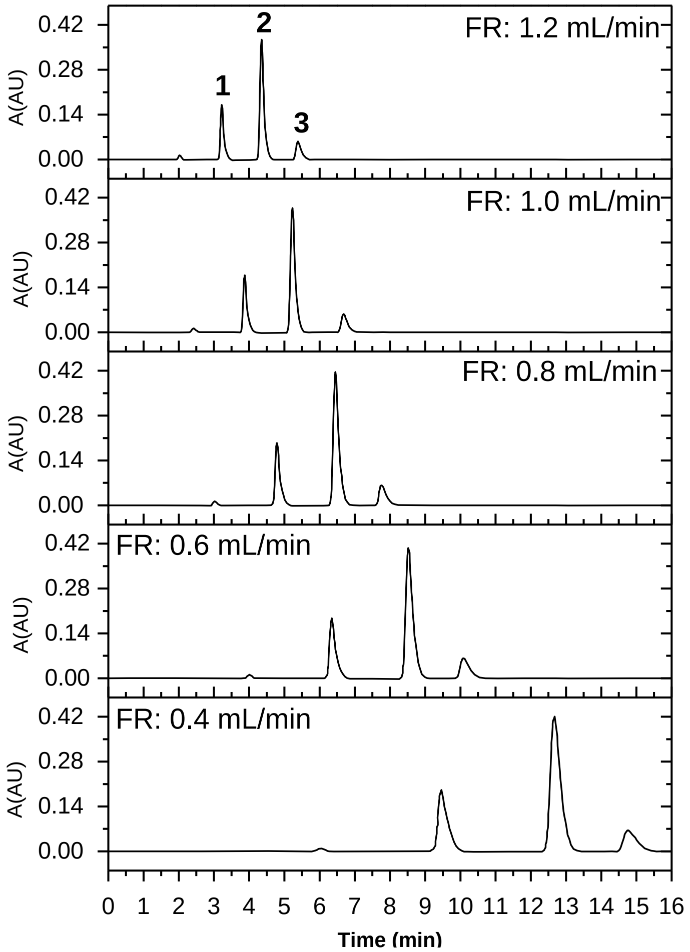


**Fig. S6.** Chromatograms obtained for several flow rates (FR) of the mobile phase using the selected 40% MeOH and pH 4.5 (1: paracetamol; 2: caffeine; 3: tramadol). Other parameters as in Fig. 1 (AU: arbitrary units).

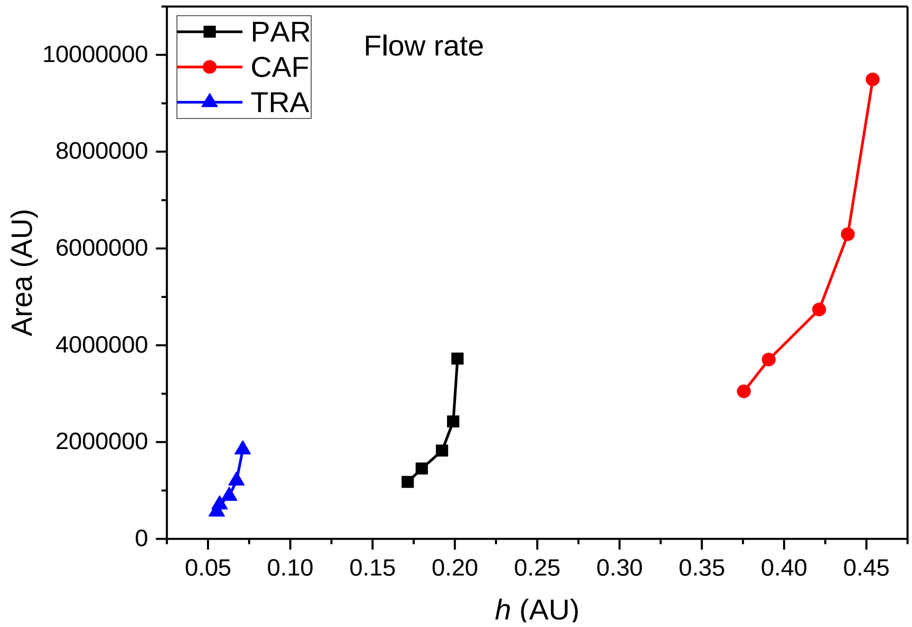


**Fig. S7.** Peak area against peak height for the three analytes and five flow rate values (AU: arbitrary units).

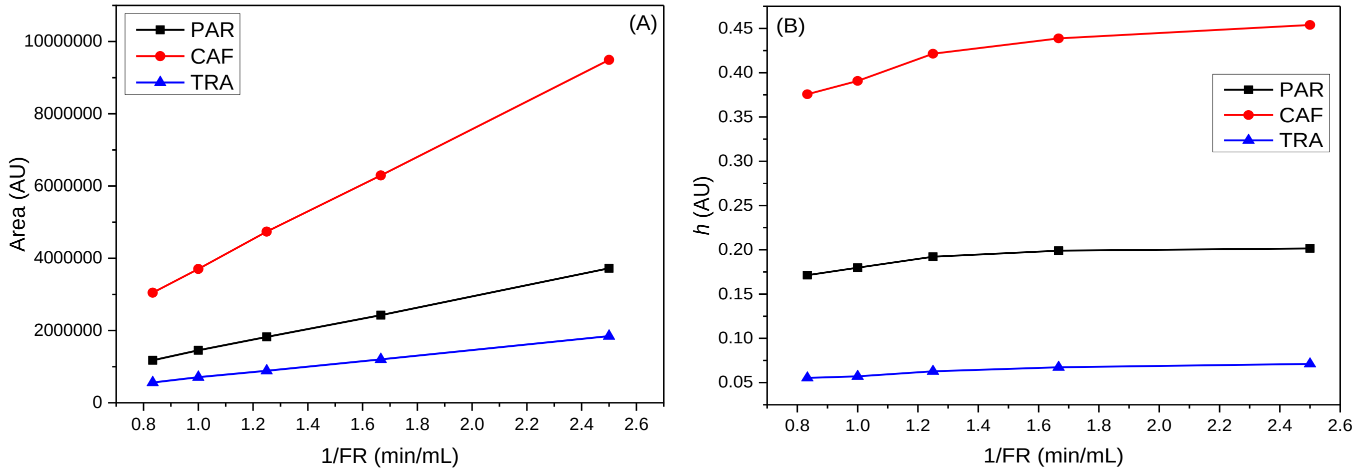


**Fig. S8.** (A) Peak area and (B) peak height against the inverse of the flow rate for the three drugs (AU: arbitrary units).

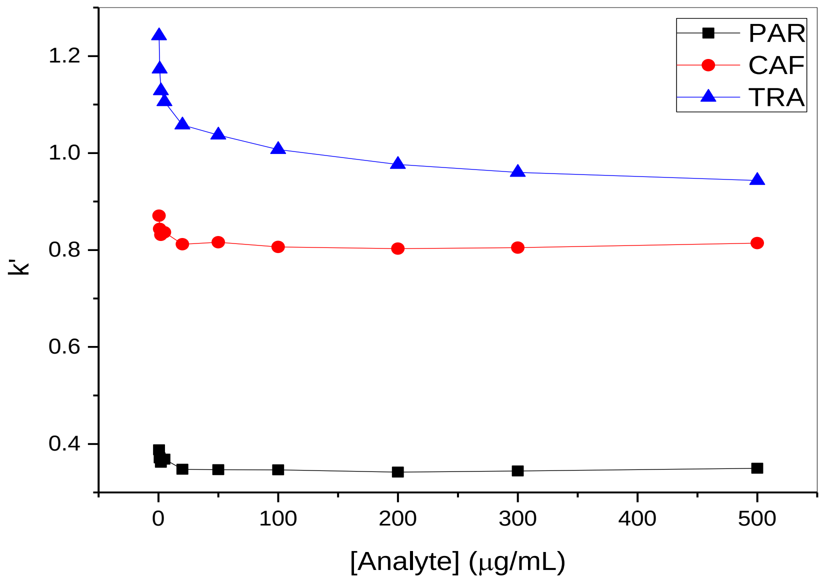


**Fig. S9.** Capacity factor against the analyte concentration for the three drugs.

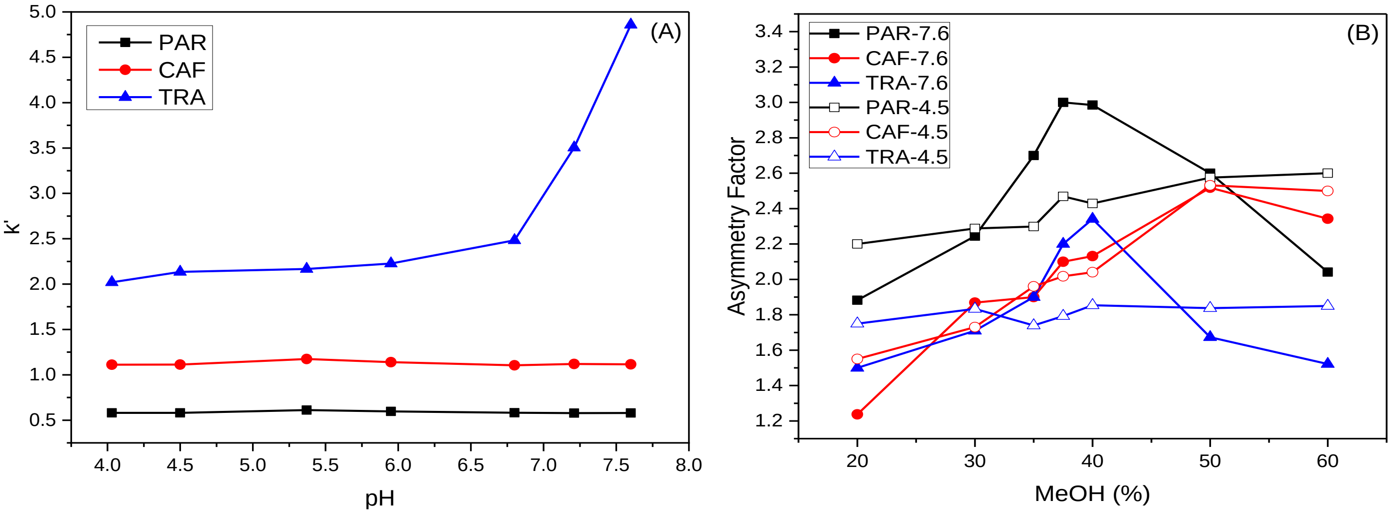


**Fig. S10.** Plots of (A) the capacity factor *vs.* pH and (B) the asymmetry factor *vs.* composition of the mobile phase at two pH values.

**Paracetamol**

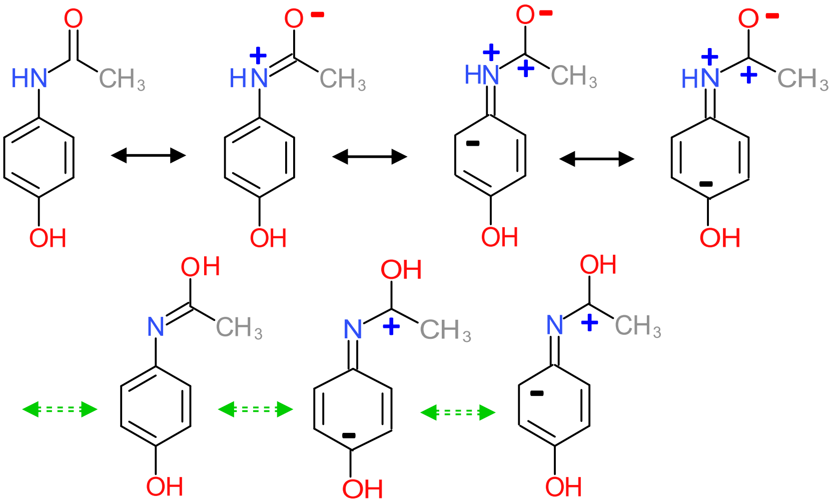

**Caffeine**

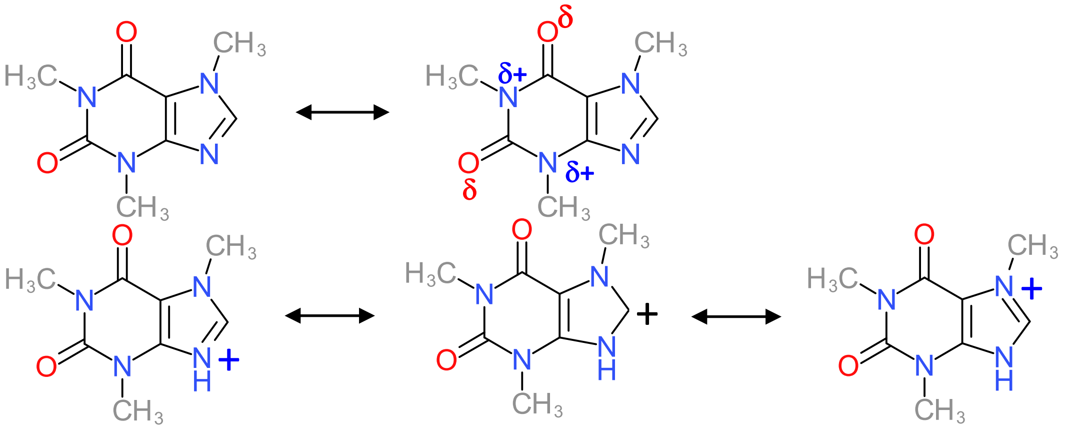

**Tramadol**

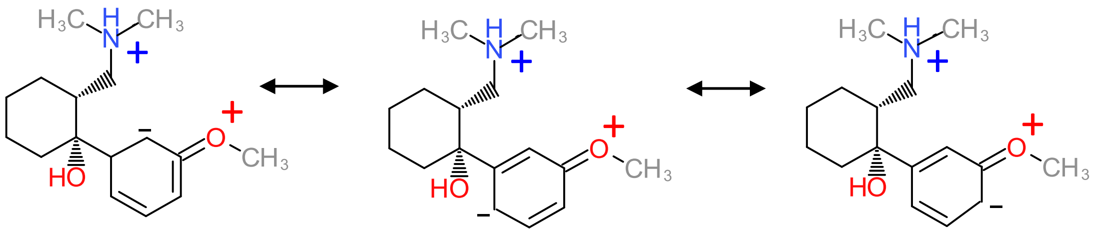

**Fig. S11.** Resonant structures for paracetamol (for two tautomers), caffeine (neutral and protonated forms) and tramadol (protonated form).

**Paracetamol**

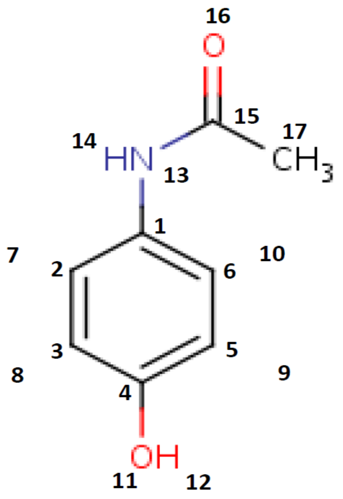


**Caffeine**

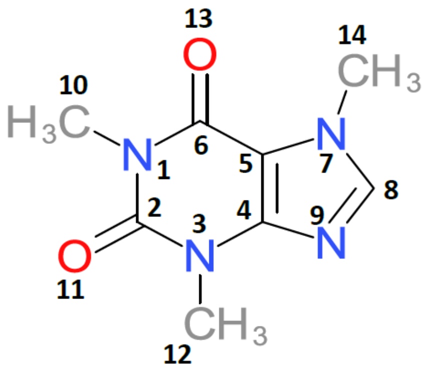


**Tramadol hydrochloride**

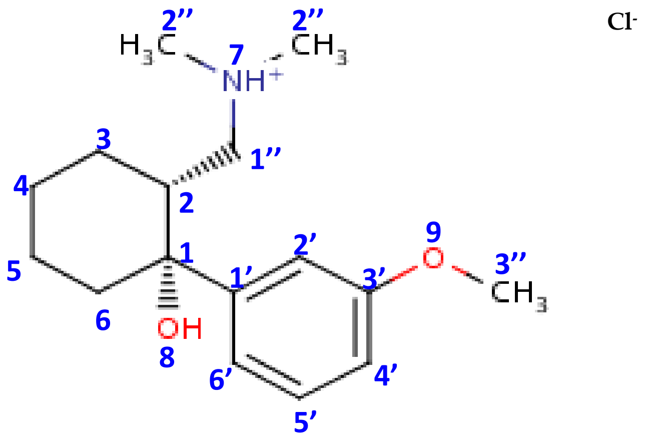


**Fig. S12.** Numeration of the carbon atoms.
